# Reviewed article: Souza ASS, Cavalcante JR, Proença R, Rodrigues IA, Frank CHM, Freitas DRC, Filho EBB, Maciel EL, Garcia MHO. History of the implementation of public health emergency management in Brazil. Epidemiol Serv Saude. 2025:34;e20240498

**DOI:** 10.1590/S2237-96222025v34e20240498.a

**Published:** 2025-05-02

**Authors:** Nathan Aratani

**Affiliations:** Universidade Federal de Mato Grosso do Sul, Instituto Integrado de Saúde

**Table:** 

Rating	Excellent	Good	Average	Below average	Poor
Interest		✓			
Quality			✓		
Originality			✓		
Overall			✓		

**Table:** 

Questionnaire	Yes	No	Not applicable
Does the manuscript contain new and significant information to justify publication?	✓		
Does the Abstract (Summary) clearly and accurately describe the content of the article?	✓		
Is the problem significant and concisely stated?	✓		
Are the methods described comprehensively?	✓		
Are the interpretations and conclusions justified by the results?	✓		
Is adequate reference made to other work in the field?	✓		

## Manuscript Structure:

Length of article is: Adequate

Number of tables is: Adequate

Number of figures is: Adequate

Please state any conflict(s) of interest that you have in relation to the review of this paper (state “none” if this is not applicable): None

## Comments

Revisões maiores:

1. No objetivo é interessante delimitar que pretende descrever o histórico de implementação no âmbito federal do SUS, uma vez que nos resultados há inclusão apenas de estratégia nacional. Estados e municípios já poderiam ter implementados iniciativas locais de vigilância e respostas, escopo que não foi contemplado pelo método da pesquisa (revisão narrativa);

2. Reveja os métodos empregados, com atenção para as ferramentas de busca bibliográfica utilizadas. Há citação do uso de base de dados da BIREME, indicação que pode estar equivocada por não ser uma base de dados, considerando que é uma associação que congrega institutos de pesquisa e bases de dados.

Revisões menores:

3. Seria interessante citar qual foi o critério para inclusão dos artigos nos resultados do trabalho.

4. Citar qual o critério para inclusão dos artigos e documentos e a delimitação pelo período a partir de 1991.

5. Na discussão explique as implicações práticas das descobertas, discutindo sua relevância para as políticas de saúde ou gestores a nível local e estadual.”

